# HIV-positive demonstrate more salt sensitivity and nocturnal non-dipping blood pressure than HIV-negative individuals

**DOI:** 10.1186/s40885-020-00160-0

**Published:** 2021-01-15

**Authors:** Sepiso K. Masenga, Annet Kirabo, Benson M. Hamooya, Selestine Nzala, Geoffrey Kwenda, Douglas C. Heimburger, Wilbroad Mutale, John R. Koethe, Leta Pilic, Sody M. Munsaka

**Affiliations:** 1 School of Medicine and Health Sciences, HAND Research Group, Mulungushi University, Livingstone, Zambia; 2 School of Health Sciences, Department of Biomedical Sciences, University of Zambia, Lusaka, Zambia; 3 Vanderbilt Institute for Global Health and Department of Medicine, Vanderbilt University Medical Center, Nashville, TN USA; 4 School of Public Health, University of Zambia, Lusaka, Zambia; 5 School of Medicine, University of Zambia, Lusaka, Zambia; 6 Faculty of Sport, Health and Applied Science, St. Mary’s University, Twickenham, London, UK

**Keywords:** Salt sensitivity, Salt, Hypertension, Nocturnal blood pressure dipping

## Abstract

**Background:**

High dietary salt and a lack of reduced blood pressure (BP) at night (non-dipping) are risk factors for the development of hypertension which may result in end-organ damage and death. The effect of high dietary salt on BP in black people of sub-Saharan Africa living with HIV is not well established. The goal of this study was to explore the associations between salt sensitivity and nocturnal blood pressure dipping according to HIV and hypertension status in a cohort of adult Zambian population.

**Methods:**

We conducted an interventional study among 43 HIV-positive and 42 HIV-negative adults matched for age and sex. Study participants were instructed to consume a low (4 g) dietary salt intake for a week followed by high (9 g) dietary salt intake for a week. Salt resistance and salt sensitivity were defined by a mean arterial pressure difference of ≤5 mmHg and ≥ 8 mmHg, respectively, between the last day of low and high dietary salt intervention. Nocturnal dipping was defined as a 10–15% decrease in night-time blood pressure measured with an ambulatory blood pressure monitor.

**Results:**

The median age was 40 years for both the HIV-positive and the HIV-negative group with 1:1 male to female ratio. HIV positive individuals with hypertension exhibited a higher BP sensitivity to salt (95%) and non-dipping BP (86%) prevalence compared with the HIV negative hypertensive (71 and 67%), HIV positive (10 and 24%) and HIV-negative normotensive (29 and 52%) groups, respectively (*p* < 0.05). Salt sensitivity was associated with non-dipping BP and hypertension in both the HIV-positive and HIV-negative groups even after adjustment in multivariate logistic regression (< 0.001).

**Conclusions:**

The results of the present study suggest that high dietary salt intake raises blood pressure and worsens nocturnal BP dipping to a greater extent in hypertensive than normotensive individuals and that hypertensive individuals have higher dietary salt intake than their normotensive counterparts. Regarding HIV status, BP of HIV-positive hypertensive patients may be more sensitive to salt intake and demonstrate more non-dipping pattern compared to HIV-negative hypertensive group. However, further studies with a larger sample size are required to validate this.

**Supplementary Information:**

The online version contains supplementary material available at 10.1186/s40885-020-00160-0.

## Background

High dietary salt intake is associated with the development of hypertension, kidney disease and cardiovascular disease (CVD), which contribute to mortality and morbidity due to myocardial infarction, renal failure, and stroke [1]. In most countries around the world estimated dietary intake of salt is on average 9–12 g per day, exceeding the World Health Organisation’s (WHO) recommended intake of less than 5 g per day [1, 2]. In experimental models and human studies, elevated salt intake increases inflammation which contributes to the development of hypertension and target organ damage [3–6]. The blood pressure (BP) response to dietary salt is heterogenous; while some individuals display a salt-induced change in BP and are considered salt-sensitive, salt-resistant individuals do not exhibit such change [7, 8]. Salt resistance and salt sensitivity are defined by a mean arterial pressure (MAP) difference of ≤5 mmHg and ≥ 8 mmHg respectively, when shifting from low- to a high-salt diet [7, 8]. Salt sensitivity predicts adverse CVD outcomes and its association with non-dipping of BP exacerbates the outcomes [9, 10]. Dipping is a physiological phenomenon where a 10–15% nocturnal drop (“dipping”) in BP is experienced during night time when a person is sleeping [9]. Hypertension is more prevalent among people living with HIV (PLWH) when compared with HIV-negative persons [11]. The factors leading to hypertension in HIV even with viral suppression from antiretroviral therapy (ART) [12], are multifactorial and may include the effects of innate and adaptive immune activation on vascular tone which could be exacerbated by dietary salt intake. However, the synergistic effects of a high salt diet and HIV infection with ART are not well established. There is a paucity of literature for sub-Saharan Africa where hypertension and HIV burden is the highest [13, 14], that explain the effect of salt on BP in PLWH contrasting with the HIV-negative individuals. The goal of this study was to explore the associations between salt sensitivity and nocturnal blood pressure dipping according to HIV and hypertension status in a cohort of adult Zambian population.

## Methods

### Study design and setting

We conducted a three-week interventional study among PLWH and an HIV-negative control group at Livingstone Central Hospital, Livingstone, Zambia. Hypertensive and normotensive participants were matched (1:1) for age and sex in both the HIV-positive and HIV-negative groups.

### Eligibility criteria

This study included PLWH with or without hypertension seen for routine health evaluations and a control group of HIV-negative adults. HIV-negative participants without hypertension were selected from volunteer health workers and those that attended routine medical examinations. We excluded those with other co-morbidities such as diabetes, tuberculosis, cancer, kidney disease and existing cardiovascular disease and HIV-positive individuals with known opportunistic infections. The diagnosis of hypertension was based on history of antihypertensive medication usage. All participants who participated were taken off medication for the period of the intervention.

### Study procedure and data collection

In the first week (7 days), the salt deprivation phase, participants were instructed not to put any salt in their food during preparation and during meals and to avoid using any processed foods. They recorded their dietary intake using a 24-h recall form (Additional file 1). In the second week, they were instructed to follow the World health organisation (WHO)/ American Heart Association recommended low salt (4 g sodium chloride/day = 1, 576 mg sodium/day), and in the third week, high salt (9 g sodium chloride/day = 3546 mg of sodium/day) was formulated by supplementing the low salt diet with additional 5 g of sodium chloride/day in the form of sodium chloride tablets. The sodium chloride tablets were manufactured from the research consolidated midland corporation division, New York, USA. Each tablet weighed one (1) gram and contained 394 mg of sodium. Participants crushed the tablets onto the portion of their meals and ingested the remainders with water. Blood pressure (BP) was measured every day for six consecutive days by the participant using a home BP machine on three occasions: at waking up, midday and in the evening before sleeping. The BP changes for low salt were calculated as previously described elsewhere [15] as BP on days 5 to 7 of low salt minus baseline as well as average BPs on the last day of each phase; those for high salt (9 g/day) were calculated as BP after high salt minus that after low salt. For 24-h BP monitoring and dipping status measurements, we used an ambulatory blood pressure monitor model ABPM50 (Contec, USA). Ambulatory BP was measured twice: on the last day of low- and high-salt diet. During the day, the interval for BP measurement was every 15 min and during night time every 30 min. The night time interval was set based on participant’s advice on the time they planned to go to bed. Day and night-time were determined by diary records of sleep and wake times taken by the participants. All readings recorded were viewed to detect any erroneous readings shown by extreme indeterminate peaks. Dipping was defined as ≥10% decrease in nocturnal BP. Participants’ mean arterial pressure (MAP) difference between low- and high-salt diet was used to define salt sensitivity (MAP≥8 mmHg) and salt resistance (MAP≤5 mmHg) [7, 8]. Three individuals with MAP difference of 6–7 mmHg were excluded. 24-h sodium urine collections were assayed at the end of each week to ensure compliance with salt intake as shown in Additional file 1. A 24-h urine sample was also collected prior to the study interventions to estimate dietary sodium intake. Participants collected their first urine sample after voiding the first morning urine which is assumed to be from the previous day’s intake. They continued collecting urine into the container until the next day with the last sample being the first urine sample after awaking from their sleep. Nocturnal urine samples were collected by the participants into a separate container during each time they awoke from their sleep. This sample, having been aliquoted, was analysed and poured back into the 24-h urine container. The urine in the 24-h urine container was mixed before being aliquoted and analysed. Urine volumes were checked to ensure they were more than 500 ml. Sodium concentrations in urine was measured using Ion-selective electrode technology with the humalyte plus 3 (Human diagnostics). More detail is available in the study protocol we previously published [16] with a slight adjustment on low salt where we used 4 g instead of 2.3 g in tandem with WHO recommendations.

### Sample size

As we previously described [16] and based on similar studies [7, 8, 17], we estimated a minimum of 21 hypertensives and 21 normotensives for PLWH and an equal sample size for the HIV negative group. Total sample size was therefore 85.

### Data analysis

We used descriptive statistics (medians, frequencies) to describe the data. As data was not approximately normally distributed, we used non-parametric tests when testing for associations and comparing proportions. Wilcoxon matched-pairs signed-rank test was used to compare median BPs for repeated measures between low- and high-salt diets. A Kruskal-Wallis test was used to compare medians of continuous variables such as age, BMI between the salt sensitive and salt resistant groups segregated by HIV status. Chi-square test with adjusted residuals for associations between salt sensitivity and the independent categorical variables such as sex (gender), hypertension, dipping and HIV status was used. McNemar’s test was used to compare differences between dippers and non-dippers on low- and high-salt diet. Univariate and multivariable logistic regression were used to determine the factors associated with salt sensitivity. Apart from HIV status, only variables that were significant in univariate analysis were included in the multivariate model. A *p*-value < 0.05 was used to determine a significant finding.

## Results

**Fig. 1:**
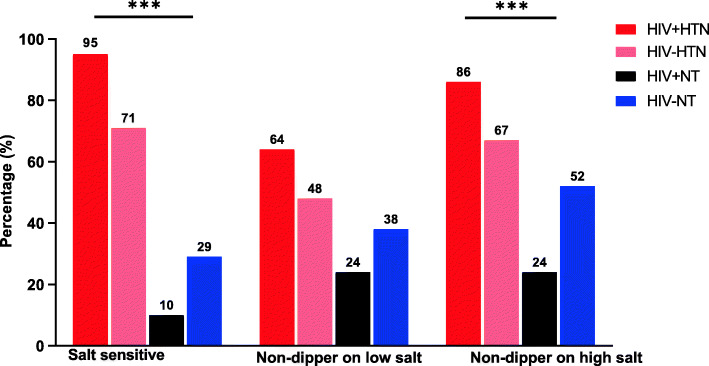
Prevalence of salt sensitivity and non-dipping blood pressure on low and high salt diets in the groups. The prevalence of salt sensitivity, non-dipping blood pressure on low and high salt diets was highest among the HIV positive hypertensive participants (HIV + HTN) followed by the HIV negative hypertensive participants (HIV-HTN) with these two groups sequentially exhibiting the highest adjusted residuals on chi-square post hoc test. HIV + NT, HIV positive normotensive participants; HIV-NT, HIV negative normotensive participants. ***p < 0.001. Chi-square test used with adjusted residuals (not shown)

**Fig. 2:**
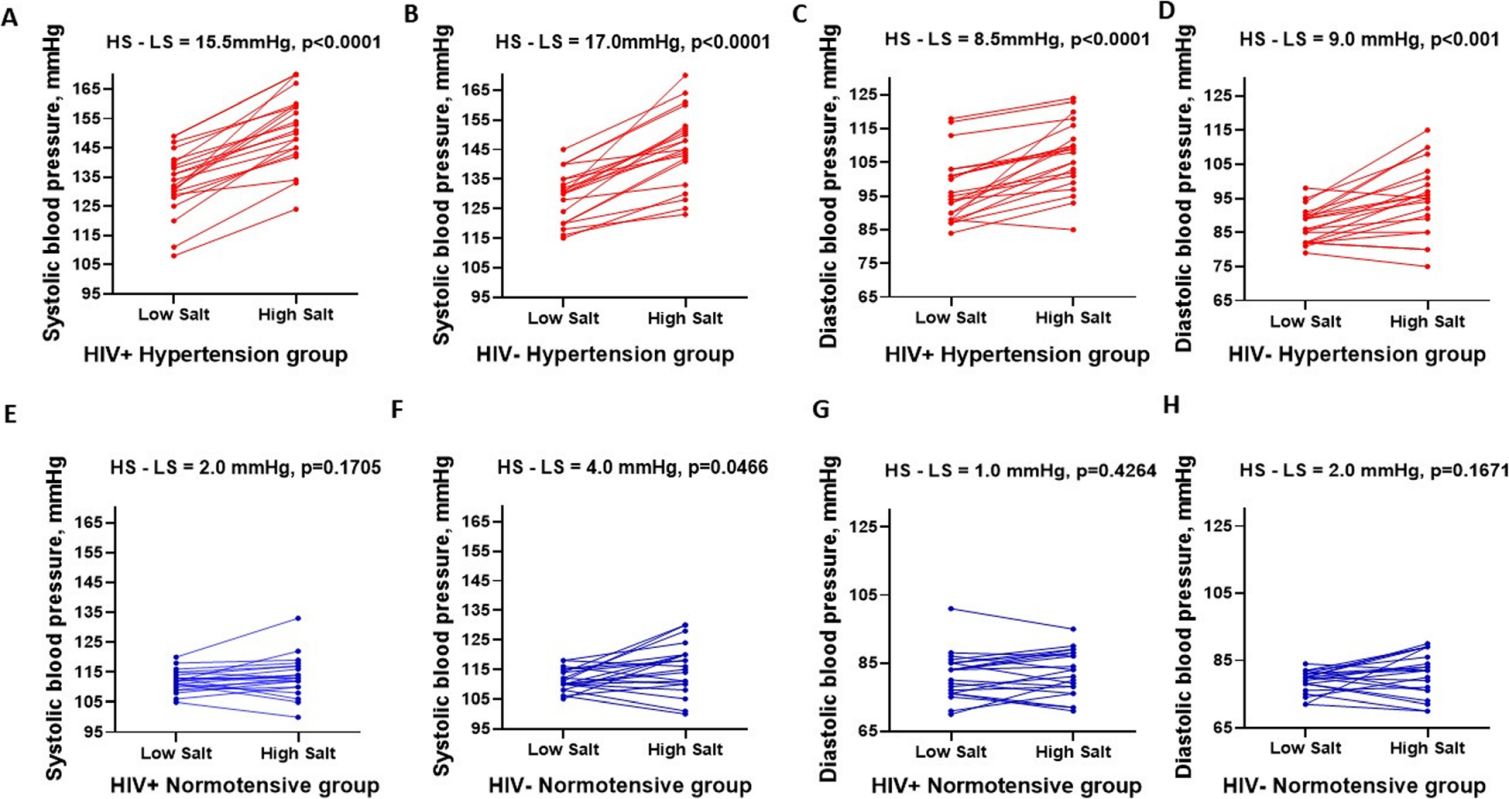
Blood pressure change in HIV positive and HIV negative groups between low and high salt diets. The hypertensive had a median systolic blood pressure (SBP) and diastolic blood pressure (DBP) change of **a** 15.5 mmHg and **c** 8.5 mmHg (*p* < 0.0001) in the HIV positive group while SBP and DBP change in HIV negative group was **b** 17.0 mmHg and **d** 9.0 mmHg, (*p* < 0.001). Normotensive groups exhibited the least SBP and DBP changes of **e** 2.0 mmHg (*p* = 0.1705) and **g** 1.0 mmHg (*p* = 0.4264) in the HIV positive group and **f** 4.0 mmHg (*p* = 0.0466) and **h** 2.0 mmHg (*p* = 0.1671) in the HIV negative group. Wilcoxon matched-pairs signed-rank test used. HS, high salt; LS, low salt

The study consisted of 85 participants comprised of 43 HIV-positive and 42 HIV-negative individuals with equal sex and age distribution (Table 1). Among the HIV positive participants, median (interquartile range) age of salt sensitive and salt resistant individuals was 40 (39, 42) and 41 (38, 42) years, respectively. The median body mass index did not differ significantly between groups. The salt sensitive and salt resistant HIV negative participants had a median (interquartile range) age of 39 (37, 41) and 40 (37, 43) years, respectively. The distribution of salt sensitivity was significantly different by hypertension and HIV status (Fig. 1, Table 1). Hypertensives had a significant rise in both systolic and diastolic blood pressure (*P* < 0.001), compared with normotensives who showed no significant change (Fig. 2). Among the hypertensive, 95% of the HIV positive and 71% of the HIV negative participants were salt sensitive (Fig. 1, Table 1). For normotensives only 10% of the HIV positive and 29% of the HIV negative were salt sensitive (Fig. 1, Table 1). The prevalence of non-dipping blood pressure on low-salt diet among the HIV positive and HIV negative hypertensive and normotensive was 64, 48, 24 and 38% respectively (Fig. 1). On high-salt diet, the prevalence of non-dipping blood pressure among the HIV positive and HIV negative hypertensive and normotensive was 86, 67, 24 and 52% respectively.

**Table 1:** Salt sensitivity and socio-demographic, clinical factors in the HIV positive and HIV negative groups

	HIV positive participants		HIV Negative participants		
	Salt sensitive n, 23	Salt resistant n, 20	Salt-sensitive n, 21	Salt-resistant n, 21	***p***-value
**Age,** *median (interquartile range)*	40 (39, 42)	41 (38, 42)	39 (37, 41)	40 (37, 43)	0.38
**Sex***, n(%)*					
*Male*	12 (31.6)	8 (21.0)	9 (23.7)	9 (23.7)	0.56
*Female*	11 (23.5)	12 (25.5)	12 (25.5)	12 (25.5)	
**Body mass index*,*** *kg/m*^*2*^	24 (20, 27)	22 (19, 25)	25 (23, 29)	23 (21, 25)	0.07
**BP status,** *n(%)*					
***Hypertensive***	21 (48.8)	1 (2.3)	15 (34.9)	6 (14.0)	**< 0.001**
*Adjusted residual*	4.57	−4.66	2.20	2.33	
***Normotensive***	2 (4.8)	19 (45.2)	6 (14.3)	15 (35.7)	
*Adjusted residual*	−4.57	4.66	−2.20	2.33	
**Dipping status on low-salt,** *n (%)*					
***Dipper, ≥10%***	8 (16.7)	16 (33.3)	8 (16.7)	16 (33.3)	**0.002**
*Adjusted residual*	−2.46	2.43	−1.96	2.10	
***Non-dipper, < 10%***	15 (40.5)	4 (10.8)	13 (35.1)	5 (13.5)	
*Adjusted residual*	2.46	−2.43	1.96	− 2.10	
**Dipping status on high-salt,** *n (%)*					
***Dipper, ≥10%***	2 (5.6)	17 (47.2)	2 (5.6)	15 (41.7)	**< 0.001**
*Adjusted residual*	−3.82	4.41	−3.51	3.11	
***Non-dipper, < 10%***	21 (42.9)	3 (6.1)	19 (38.8)	6 (12.2)	
*Adjusted residual*	3.82	−4.41	3.51	−3.11	

Row percentage used. Kruskal-Wallis test and Chi-square test used. *P*-value less than 0.05 are in bold

### Salt sensitivity is associated with hypertension and non-dipping blood pressure

Salt sensitivity was associated with hypertension and dipping status across the groups (Table 1). Salt sensitivity among the hypertensive was more pronounced in the HIV positive group compared with the HIV negative as shown by the adjusted residuals (Table 1). This trend was similar for non-dipping blood pressure also. The percentage of non-dippers increased by approximately 26 and 28% in HIV positive and HIV negative salt-sensitive groups respectively, when participants were shifting from low- to high-salt diet.

To determine if salt sensitivity was a factor for shifting from a dipper to non-dipper, we compared the number of dippers vs. non-dippers on low- vs high-salt diet in the salt-resistant group and salt-sensitive group (Table 2). There was a significant change in the number of dippers vs. non-dippers when shifting from low- to high-salt diet only in salt-sensitive group (*P* = 0.031), regardless of HIV status.

**Table 2:** Dippers vs non dippers on low- vs high-salt diet in the HIV positive and HIV negative

	low-salt HIV positive, *n (%)*		***p***-value	low-salt HIV negative, *n (%)*		***p***-value
	*Dipper*	*Non-dipper*		*Dipper*	*Non-dipper*	
**Salt-sensitive group**						
high-salt, *n (%)*						
*Dipper*	2 (25)	0 (0)	**0.031**	2 (25.0)	0 (0.0)	**0.031**
*Non-dipper*	6 (75)	15 (100)		6 (75.0)	13 (100.0)	
**Salt-resistant group**						
high-salt, *n (%)*						
*Dipper*	16 (100)	1 (25)	> 0.99	15 (93.8)	0 (0.0)	> 0.99
*Non-dipper*	0 (0)	3 (75)		1 (6.3)	5 (100.0)	

Row percentage. McNemar’s test used

In multivariate logistic regression analysis, non-dipping blood pressure on high salt diet and hypertension remained significantly associated with salt sensitivity (*p* < 0.001) while HIV status and non-dipping blood pressure on low salt diet were not significantly associated with salt sensitivity (Table 3).

**Table 3:** Association between Salt sensitivity and each clinical-physiological characteristic in the study population (HIV positive combined with HIV negative)

Variable	Odds Ratio OR (95%CI)	***p***-value	Adjusted Odds Ratio AOR (95%CI)	***p***-value
Non-dipping blood pressure on low-salt	6.22 (2.37, 16.27)	**< 0.001**	0.17 (0.01, 2.18)	0.17
Non-dipping blood pressure on high-salt	35 (10, 126)	**< 0.001**	337 (14, 7791)	**< 0.001**
Body mass index	1.12 (1.01, 1.25)	**0.031**	0.92 (0.74, 1.15)	0.48
HIV positive	1.15 (0.49, 2.69)	0.74	1.39 (0.26, 7.44)	0.70
Hypertension	21 (7, 66)	**< 0.001**	72 (6, 772)	**< 0.001**

*P*-value less than 0.05 are in bold

### Sodium excretion in HIV-positive and HIV-negative groups

**Fig. 3:**
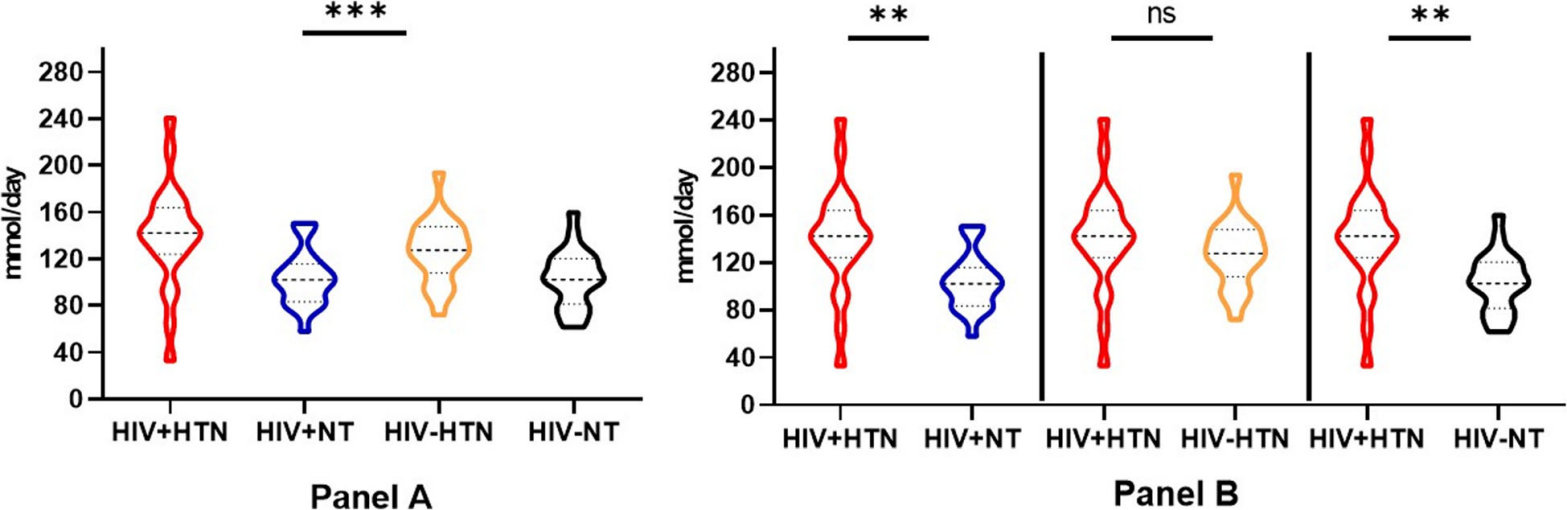
24-h urine sodium excretion prior to the intervention in the groups. Sodium excretion was different across groups (Panel **a**, *p* = 0.0003). As shown in panel **b**, HIV positive hypertensive (HIV + HTN) excreted higher sodium in urine compared with HIV positive normotensive (HIV + NT) and HIV negative normotensive (HIV-NT) groups (*p* = 0.001) but not with the HIV negative hypertensive group (*p* = 0.89). Median and interquartile range for the groups in mmol/day: HIV + HTN 142 (124, 164), HIV + NT 102 (83, 115), HIV-HTN 127 (108, 147), HIV-NT 102 (81, 120). The overall mean (SD) sodium excretion in the population was 119 ± 35 mmol/day (2737 ± 805 mg/day of sodium or ≈ 6.8 ± 2 g salt/day). The minimum and maximum sodium secreted was 759–7, 920 mg per day (≈2 – 19 g salt). ***p < 0.001, **p < 0.01. Kruskal-Wallis (panel **a**) followed by Dunn’s multiple comparisons test (panel **b**)

24-h excretion of sodium prior to the interventions differed by hypertension status (Fig. 3). HIV positive hypertensive participants excreted more sodium in their urine compared to the HIV positive and HIV negative normotensive (*p* < 0.01). The amount of sodium excreted between the HIV positive hypertensive and the HIV negative hypertensive participants did not differ significantly. The estimated average (standard deviation) dietary sodium intake in the study population prior to the dietary interventions was 2, 737 ± 805 mg per day equivalent of 6.8 ± 2 g salt/day (Additional file 1). Average sodium (salt) intake on low- and high-salt was 1, 766 ± 170 mg/day (4.4 ± 0.4 g of salt/day) and 5, 021 ± 1437 mg/day (12.6 ± 3.6 g of salt/day).

Nocturnal secretion of sodium on low- and high-salt diet was not significantly different between the HIV positive hypertensive and HIV negative hypertensive but only between hypertensives and normotensives regardless of HIV status (Additional file 1).

## Discussion

The goal of this study was to explore the associations between salt sensitivity and nocturnal blood pressure dipping according to HIV and hypertension status in a cohort of adult Zambian population. Ninety five percent (95%) of HIV positive hypertensive individuals had their blood pressure significantly raised by high salt intake compared with 71% HIV negative hypertensive, 10% HIV positive normotensive and 29% HIV negative normotensive participants (Fig. 1, Table 1, *P* < 0.05). The trend was similar for non-dipping blood pressure. The ‘salt-sensitive’ phenotype was responsible for the BP modulation and consequently, dipping status. 24-h excretion of sodium in urine prior to the intervention was higher in hypertensives compared to normotensives regardless of HIV status (Fig. 3, *p* < 0.05).

Although the prevalence of SSH was high in both HIV positive (95%) and HIV negative (71%) groups, this correlates with other studies suggesting several elusive mechanisms such as genetic predisposition [18], among others, that link the ‘black race’ with more susceptibility to SSH [19, 20]. Specifically, these mechanisms include genetic variations affecting activity of sodium transport proteins, kidney damage mediated by inflammation, the aldosterone mineralocorticoid receptor pathway and neuronal alterations, among others [21]. Among the neuro-endocrine factors involved in the salt sensitivity of blood pressure (SSBP) are the renin-angiotensin-aldosterone system (RAAS), the sympathetic nervous system, natriuretic peptides, insulin, leptin and various endothelial effectors with endocrine and/or paracrine activity. Most of these affect the regulation of tubular sodium and water reabsorption and, thus, volume homeostasis [6, 15, 22–24]. Another factor that may exacerbate hypertension in HIV-infected people is that greater CD8 activation is associated with less arterial distensibility and flow-mediated dilation (indicative of endothelial dysfunction) [25]. These data suggest that salt sensitivity phenotype is a risk factor for the development of hypertension and may worsen already existing hypertension among PLWH.

The prevalence of SSH in the general population is approximately 50% in hypertensive and 25% in the normotensive and salt sensitivity has been linked to endothelial dysfunction and adverse cardiovascular events [22, 26]. The prevalence of salt sensitivity among the hypertensive was high for both HIV positive and HIV negative participants in our study. The HIV positive hypertensive demonstrated more salt sensitivity compared with the HIV negative hypertensive. It is, however, difficult to make direct comparisons to other studies due to differences in protocols [26–28] used to diagnose salt sensitivity. Moreover, the main aim of this study was not to determine salt sensitivity prevalence but to explore the effects of salt loading on nocturnal BP dipping. These results should therefore be regarded with caution and we recommend a randomized crossover intervention with dietary sodium employed in order to explore the true prevalence of salt sensitivity.

Control mechanisms for blood pressure are many including arterial baroreceptors and chemoreceptors, the central nervous system ischemic response, the renin-angiotensin system, and capillary fluid shift, however, all these mechanisms are overridden by pressure–natriuresis [9], a concept where a rise or fall in blood pressure correlates positively with natriuresis. Excretion of sodium follows a diurnal rhythm where excretion reaches a maximum during the day and a minimum at night during sleep owing to a drop in blood pressure (pressure-natriuresis concept). However, our results suggest that low and high salt intake was associated with higher nocturnal natriuresis in both HIV-positive and HIV-negative hypertensive individuals. Considering a small sample size, this result should be evaluated in a larger study population. Nevertheless, this phenomenon is not uncommon, as it has been previously reported [9]. Normotensive salt-resistant individuals have the expected nocturnal decrease in sodium excretion. The underlying mechanism behind this is the increase in blood pressure seen in hypertensive and in salt sensitivity.

**Fig. 4:**
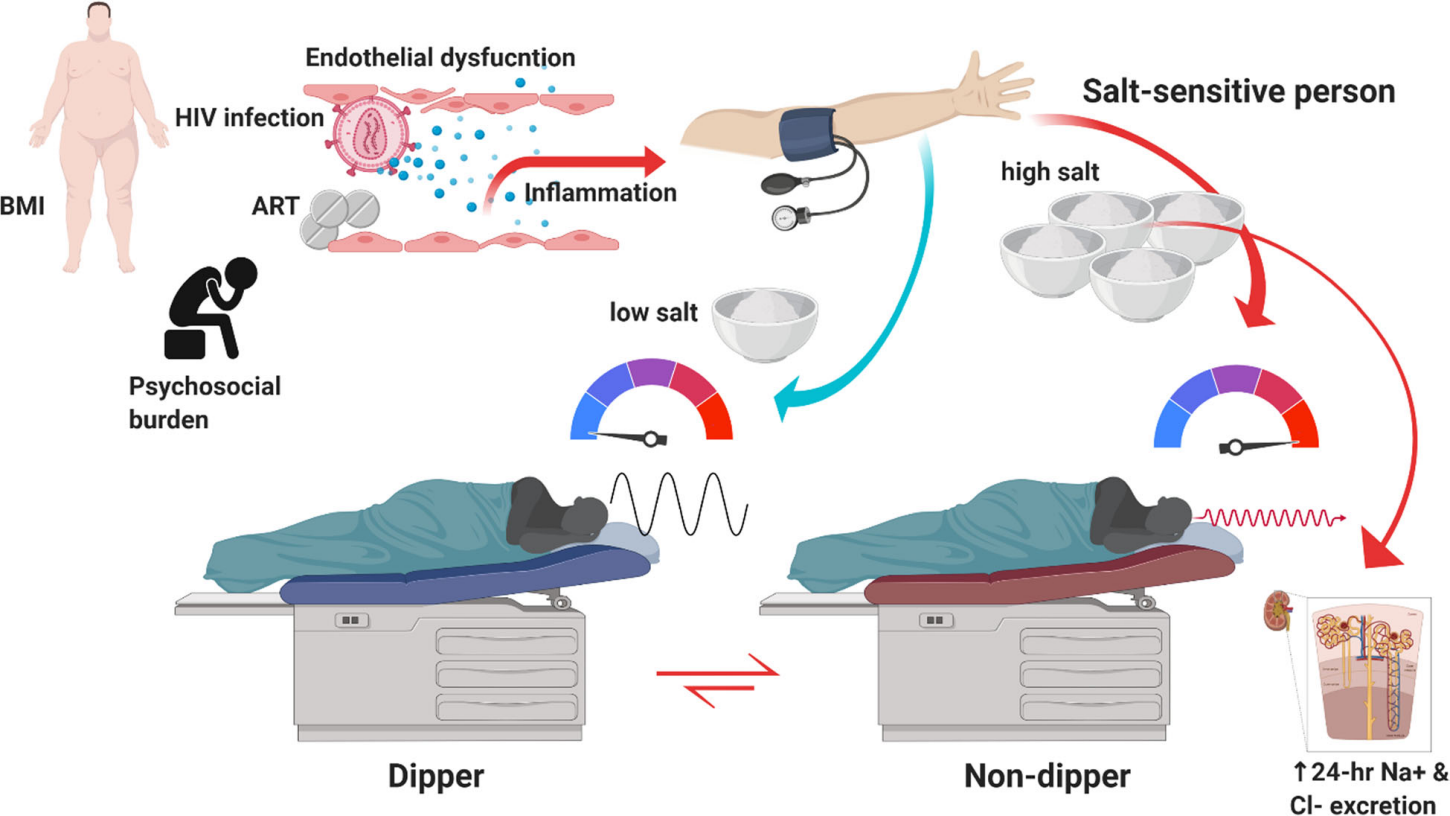
Correlates of dipping status in HIV. Dipping status is affected by salt-intake, inflammatory adiposity in high Body mass index (BMI), HIV-infection, antiretroviral therapy (ART) effects of propagating endothelial dysfunction, psychosocial burden and chronic inflammation. All these factors modulate the cardiovascular system and mechanisms involved in dipping. Urine excretion of sodium and chloride is high in salt-sensitive persons as a mechanism of reducing high blood pressure. A salt-sensitive person may switch from a dipper to non-dipper on both low- and high-salt diet

Dipping is a physiological phenomenon where blood pressure declines > 10% during night sleep. For a non-dipper, night systolic blood pressure decline does not exceed 10% that of day. Non-dipping status has been associated with target organ damage, left ventricular hypertrophy, microalbuminuria and cerebrovascular disease [9]. Non-dipping in our study was associated with increased salt intake and was more pronounced in salt-sensitive individuals regardless of HIV status, although PLWH had more non-dippers compared with the HIV negative on both low- and high-salt diets. Hence, dietary salt has implications for the management of hypertension. There is evidence from studies conducted in sub-Saharan Africa confirming that non-dipping blood pressure prevalence is high in PLWH [10]. Previous studies reported that an abnormal diurnal blood pressure pattern may be more common among PLWH versus HIV-negative individuals [29]. The mechanisms underlying non-dipping status in HIV contrasting that of HIV-negative are unclear. However, a plausible explanation is that chronic inflammation and HIV infection that contributes to endothelial dysfunction and further exacerbated by ART may be the underlying mechanism resulting in dysregulation of the cardiovascular rhythm responsible for dipping [30]. Other factors associated with non-dipping status in HIV include but not limited to increased levels of inflammatory biomarkers, high psychosocial burden, high prevalence of sleep disturbance, and autonomic dysfunction [29] as summarized in Fig. 4.

One of the physiological explanations for the contrasting response to salt exhibited by salt-sensitive and salt-resistant individuals may be vasodilation. Salt-sensitive individuals do not vasodilate to accommodate fluid load that follows high-salt loading hence an increase in blood pressure results whereas salt-resistant individuals vasodilate [9]. As a consequence, renal blood flow (RBF) is lowered and renal vascular resistance (RVR) increased in high-salt diets resulting in higher blood pressure.

### Study limitations and strengths

A larger sample size is required to best estimate the prevalence of SSH in PLWH and the general population, thus, we cannot generalize our findings to the general population. This is also seen by the wide confidence interval in Table 3. In addition, participants were instructed on diets and not provided meals which may result in poor adherence. Although sodium excretion on the high-salt diet was slightly higher than what was prescribed, compliance to low-salt diet was generally good, and sodium intake of participants across the two diets were consistent (Additional file 1). Unfortunately, a complete 24-h urine was not validated by urine creatinine excretion. Although completeness of urine collections was not assessed by measuring urinary creatinine excretion, all participants provided collections higher than 500 ml suggesting satisfactory collections. A strength of this study lies in the use of automated ambulatory BP monitors (ABPM) to measure 24-h BP. As this is uncommon in sub-Saharan Africa, this is likely one of the first studies exploring salt sensitivity and nocturnal dipping using ABPM in PLWH. ABPM is superior to both office and home BP monitoring due to a number of BP readings taken automatically throughout the day and as BP varies during the day, ABPM eliminates inaccuracies arising from a single reading [31].

## Conclusions

The results of the present study suggest that high dietary salt intake raises blood pressure and worsens nocturnal BP dipping to a greater extent in hypertensive than normotensive individuals and that hypertensive individuals have higher dietary salt intake than their normotensive counterparts. Regarding HIV status, BP of HIV-positive hypertensive patients may be more sensitive to salt intake and demonstrate more non-dipping pattern compared to HIV-negative hypertensive group. However, further studies with a larger sample size are required to validate this. If this is indeed the case, then HIV positive individuals with hypertension should be particularly advised to reduce their salt intake to prevent adverse effects associated with hypertension and non-dipping blood pressure.

## Supplementary Information

**Additional file 1: Table S1.** 24-h food intake record. **Table S2.** Sodium excretion on low- and high-salt diet and baseline random 24-h sodium excretion. **Table S3.** Estimated sodium intake of the study participants on low- and high-salt diet and prior to the interventions. **Table S4.** Nocturnal sodium excretion in the HIV-positive and HIV negative groups.

## Data Availability

All data generated or analysed during this study are included in this published article. For other data, these may be requested through the corresponding author.

## Abbreviations

ABPM: Ambulatory blood pressure monitor
AOR: Adjusted odds ratio
ART: Antiretroviral therapy
BMI: Body mass index
BP: Blood pressure
CVD: Cardiovascular disease
DBP: Diastolic blood pressure
HIV: Human immunodeficiency virus
IQR: Interquartile range
ISE: Ion-selective electrode
LCH: Livingstone Central Hospital
MAP: Mean arterial pressure
OR: Odds ratio
PLWH: People living with HIV
PP: Pulse pressure
RAAS: Renin angiotensin aldosterone system
RBF: Renal blood flow
RVR: Renal vascular resistance
SBP: Systolic blood pressure
SSH: Salt-sensitive hypertension
SD: Standard deviation
WHO: World health organisation
