# Supplementary material for: HIV-positive demonstrate more salt sensitivity and nocturnal non-dipping blood pressure than HIV-negative individuals
Source: Clin Hypertens. 2021 Jan 15;27:2. doi: 10.1186/s40885-020-00160-0 (PMC7809779; doi:10.1186/s40885-020-00160-0)
Supplement: Supplementary file 1 — Additional file 1: Table S1. 24-h food intake record. Table S2. Sodium excretion on low- and high-salt diet and baseline random 24-h sodium excretion. Table S3. Estimated sodium intake of the study participants on low- and high-salt diet and prior to the interventions. Table S4. Nocturnal sodium excretion in the HIV-positive and HIV negative groups. [file 40885_2020_160_MOESM1_ESM.docx]

**HIV-positive demonstrate more Salt sensitivity and nocturnal non-dipping blood pressure than HIV-negative individuals**

Sepiso K. Masenga,^1,2^ Annet Kirabo,^3^ Benson M. Hamooya,^1,4^ Selestine Nzala,^5^ Geoffrey Kwenda,^2^ Douglas C. Heimburger,^3,5^ Wilbroad Mutale,^4^ John R. Koethe,^3^ Leta Pilic,^6^ Sody M. Munsaka,^2^

^1^Mulungushi University, School of Medicine and Health Sciences, HAND research group, Livingstone, Zambia

^2^University of Zambia, School of Health Sciences, Department of Biomedical Sciences, Lusaka, Zambia

^3^Vanderbilt Institute for Global Health and Department of Medicine, Vanderbilt University Medical Center, Nashville, TN, USA.

^4^University of Zambia, School of Public Health, Lusaka, Zambia

^5^University of Zambia School of Medicine, Lusaka, Zambia

^6^St. Mary’s University, Faculty of Sport, Health and Applied Science, Twickenham, London, UK

**Corresponding Author:**

Sepiso K. Masenga, BSc, MSc, Pgcert

HAND Research Group

Mulungushi University

School of Medicine and Health Sciences

P.O Box 60009

Livingstone Central Hospital campus

Livingstone, Zambia

Telephone: +260 966 67 4774

Email: [smasenga@mu.ac.zm](mailto:smasenga@mu.ac.zm) or sepisomasenga@gmail.com

**Table S1. 24-hour food intake record**

Study ID#: Gender: Date:

| **Breakfast** | | | Time:  Place: | |
| --- | --- | --- | --- | --- |
| Name | Food description | Estimated Amount | Number and method of Salt tablet consumption | Preparation/ Ingredients |
|  |  |  |  |  |
|  |  |  |  |  |
| **Lunch** | | | Time:  Place: | |
|  |  |  |  |  |
|  |  |  |  |  |
| **Dinner** | | | Time:  Place: | |
|  |  |  |  |  |
|  |  |  |  |  |
| **Other in-between meals (indicate times for each meal)** | | | Time:  Place: | |
|  |  |  |  |  |
|  |  |  |  |  |
|  |  |  |  |  |

Name=name of the food in English or local language; Food description= Participant describes the food; Estimated Amount; Participant estimates the amount of food using a fist and palm, number of fists and number of palms dependent on type of food consumed; Number and method of Salt tablet consumption= Salt tablets consumed at each meal are indicated. Two methods of possible consumption were indicated which was by swallowing/ingesting the tablets with water and crushing the tablets to powder and adding the powder to the portion of their food on the plate. Preparation/ Ingredients=participants were to indicate if they added any processed foods or spices with salt

**Table S2. Sodium excretion on low- and high-salt diet and baseline random 24-hr sodium excretion**

|  | **HIV Positive** (median (IQR) | | **HIV Negative** (median (IQR) | |  |  |
| --- | --- | --- | --- | --- | --- | --- |
|  | hypertensive | normotensive | hypertensive | normotensive | p-value |  |
| **Low salt diet** |  |  |  |  |  |  |
| Urine Sodium, *mmol/day* | 79.1 (75.8, 85.9) | 78.1 (72.3, 80.4) | 77.3 (72.9, 79.3) | 76.7 (72.0, 78.3) | 0.09 |  |
| **High salt diet** |  |  |  |  |  |  |
| Urine Sodium, *mmol/day* | 221.9 (175.3, 270.7) | 235.4 (166.1, 285.3) | 208.8 (183.2, 250.1) | 235.8 (200.0, 264.1) | 0.56 |  |
| **24-hr sodium excretion prior to the intervention**, mmol/day | 142 (124, 164) | 102 (83, 115) | 127 (108, 147) | 102 (81, 120) | <0.001 |  |

IQR, interquartile range; Kruskal-Wallis test used. All participants excreted comparable amounts of sodium in urine during the low and high salt diets.

**Table S3. Estimated sodium intake of the study participants on low- and high-salt diet and prior to the interventions**

|  | Population, n=85  Average ± SD | Population, n=85 Average (minimum, maximum) |
| --- | --- | --- |
| **Low salt diet** |  |  |
| Sodium intake, *mg/day* | 1766 ± 170 | 1178, 2175 |
| Salt intake, g/day | 4.4 ± 0.4 | 2.9, 5.4 |
| **High salt diet** |  |  |
| Sodium intake, *mg/day* | 5021 ± 1437 | 1242, 8556 |
| Salt intake, g/day | 12.6 ± 3.6 | 3.1, 21.4 |
| **Baseline intake** |  |  |
| Sodium intake, *mg/day* | 2, 737 ± 805 | 761, 5520 |
| Salt intake, g/day | 6.8 ± 2 | 1.9 ± 13.8 |

IQR, interquartile range; SD, standard deviation. To convert sodium from mmol to mg, we multiplied mmol X 23. To convert sodium to salt, we multiplied mg of sodium by 2.5/1000.

**Table S4. Nocturnal sodium excretion in the HIV-positive and HIV negative groups**

|  | **HIV positive** | | **HIV negative** | |  |
| --- | --- | --- | --- | --- | --- |
| **Clinical characteristics** | **Hypertensive (HTN)**  **n, 22** | **Normotensive (NT)**  **n, 21** | **Hypertensive (HTN)**  **n, 21** | **Normotensive (NT)**  **n, 21** | **p-value** |
| **Nocturnal sodium excretion on low-salt diets**, mmol/l | 39 (33, 56) | 33 (29, 43) | 51 (39, 63) | 33 (22, 46) | <0.001 |
| Significantly different groups | HIV-HTN vs HIV+NT; HIV-HTN vs HIV-NT | | | | **<0.01** |
|  |  | | | |  |
| **Nocturnal sodium excretion on high-salt diets**, mmol/l | 78 (68, 90) | 65 (56, 73) | 84 (75, 93) | 76 (70, 81) | **<0.001** |
| Significantly different groups | HIV+HTN vs. HIV+NT, HIV-HTN vs. HIV+NT | | | | **<0.05** |

Row percentage used; Wilcoxon rank-sum test used. P-value less than 0.05 are in bold

Kruskal-Wallis test with Dunn’s multiple comparison
